# Performing a ‘Returnee’ in Benin City, Nigeria

**DOI:** 10.1007/s12134-022-00976-9

**Published:** 2022-07-23

**Authors:** Mariia Shaidrova

**Affiliations:** University of Tilburg, Tilburg, The Netherlands

**Keywords:** Return migration, Returnees, Labelling, Identity construction, Nigeria, Benin City

## Abstract

Over recent years, with the support of international NGOs, many thousands of irregular migrants were ‘returned’ to West Africa from Libyan detention centres. Through extensive ethnographic fieldwork with different groups of returnees in Benin City, Nigeria, I studied the establishment and evolution of the ‘returnee’ identity. Making use of labelling, social identity and performativity theories, I found that the performance of the returnee identity for Western donors, researchers and the media creates opportunities for the returnees to regain respect in their communities. Emphasising the role of performativity in identity formation, I use the metaphor of a theatrical play. Initially scripted by the EU border-externalisation policies, the return-migration play has evolved to fit in local political realities. On the frontstage, returnees were adjusting to the EU counter-migration agenda, testifying about the risks of irregular migration. Backstage, however, they kept pursuing their migration aspirations, also using the returnee identity to establish themselves in the city and gain some level of political recognition.

## Introduction

Andrew,^1^ a 25-year-old Nigerian, returned to his country from Libyan detention camps in 2017 with the support of the International Organization for Migration (hereafter, IOM). When we met in March 2020 at a government-funded fisherman training facility for returnees from Libya, Andrew saw my camera and presented himself as a cinematographer interested in the fishing industry. Upon his return, Andrew had completed the reintegration programme of the IOM and shortly after joined programmes, designed to counter irregular migration, organised by both governmental and non-governmental organisations in Benin City. Andrew was proud to be a returnee because it eventually helped him to get into the Benin Film School free of charge. Although Andrew would not describe his own experiences in Libya as ‘horrific’ but simply mentioned difficulties crossing the Sahara Desert, he contended that not everyone was as lucky. Andrew is still dreaming of joining his brother, who has managed to make a living in Spain.

In recent years, with the support of the IOM, thousands of migrants have ‘returned’ to West Africa from detention camps in Libya where they faced inhumane conditions and maltreatment (Human Rights Watch, 2019). More precisely, the IOM launched its return operation — Voluntary Humanitarian Returns (VHR) — in 2017 as part of the larger EU–IOM Joint Initiative for Migrant Protection and Reintegration, which gives migrants stranded in Libya the option to return back home and offers them reintegration assistance in the form of livelihood training and financial support. It has been described by the IOM as ‘an immediate and necessary life-saving option for thousands of people both inside and outside of detention centers’ (IOM, 2020). As a result, nearly 16,000 migrants were returned to Nigeria in the period between 2017 and 2019 (Alpes, 2020).

Although the numerous reports about human-rights abuses in the Libyan detention centres justify calling the operation ‘humanitarian’ and ‘voluntary’, it remains questionable to what extent returns can be seen as ‘voluntary’ due to the lack of alternatives (Webber, 2011). Discussing the concept of ‘voluntariness’, Olsaretti (1998) emphasises that choices are voluntary only in the case of acceptable alternatives being available. ‘Acceptable’ alternatives are not easy to conceptualise and, according to Erdal & Oeppen (2018), migrants’ options are often restricted by information received from immigration authorities and international NGOs (for instance, the UNHCR, the IOM). By shaping the options available to migrants, humanitarian returns operated by the IOM take a form of externalised migration management or control (Ashutosh & Mountz, 2011; Brachet, 2015; Triandafyllidou & Ricard-Guay, 2019). Pécoud (2013) describes such efforts as a preventive way to discipline local states and shape aspirations of potential and failed migrants. However, the EU border externalisation policies in general — and VHRs in particular — do not seem to succeed in ‘disciplining’ local governments (Rodriguez, 2019).

Anthropologists studying developing countries have long argued that policy models are transformed by those who benefit from them (Mosse & Lewis, 2006). The initial policy — in this case, the EU anti-migration agenda that has manifested itself in returns and reintegration carried out by the IOM — is translated into the everyday reality of local officials and grassroots organisations led by returnees. By translating between ‘different institutional languages’ (Mosse, 2004: 647), local brokers (i.e. post-return caregivers and the leaders of the returnee-led organisations) serve the counter-migration agenda of Western policy-makers. Simultaneously, they use this agenda to establish themselves professionally — as was illustrated in the case of Andrew, above — and fight the stigma associated with return (Alpes, 2017).

The objective of this paper is to illustrate how EU border externalisation policies politicised the returns and prepared the grounds for a new ‘returnee’ category to be established in Benin City. To understand the returnee’s position, I explore labelling and social-identity theories, especially in the context of stigma and victimisation (Dunn, 2008; Goffman, 1963; Loseke, 2001). More precisely, I look at how ‘spoiled identity’ (Goffman, 1963) can serve as a source of self-establishment. Combining these diverse theoretical perspectives and concepts, I propose to use the metaphor of a theatrical play. The EU counter-migration policies, including returns, reintegration and counter-migration awareness activities are described as a ‘scene-setting’ for performative acts. When the scene is set, the actors start performing and navigating the stage, adjusting their acts to the setting and the audience that includes ‘scene setters’ (Brussels, EU member states, UN institutions) and communities (the public). By applying this metaphor to the return-migration play in Benin City, I do not necessarily focus on the process of the scene-setting or conditions for the acting but, rather, explore how the play is being navigated by its main actors — returnees. More precisely, I look at how returnees’ engagement with the formal framework of migration governance leads to capitalisation on their material resources, skills and status, which can be interpreted as an expression of migrants’ agency.

## Politicisation of the Humanitarian Return Operations

Homecoming as a phenomenon has been addressed by academics for decades, especially in the context of transnational development (King, 2015), integration failure (Carling & Erdal, 2014), and the return of diasporas, including second-generation migrants (Vathi & King, 2011). However, in recent years, the governance of returns has received more academic attention. It is especially relevant in the context of deportations and organised ‘voluntary’ returns from the countries of origin and transit (e.g. Koser & Kuschminder, 2015; Peutz & de Genova, 2010). If the returns are managed and assisted by a third party, then naturally, the question of volition arises. Therefore, according to Kleist (2018), the most obvious way to differentiate homecoming would be by focusing on its voluntary and involuntary aspects. By involuntary return, Kleist implied deportations and evacuations where the choice is restricted and governed by circumstances that are beyond the migrant’s control. As argued by Kleist (2017), even in involuntary returns, migrants might be more welcome in their communities of origin if they do not return empty-handed. More broadly, return is considered sustainable in cases when migrants have the means to achieve something and establish themselves back home (Koser & Kuschminder, 2015). However, the funds allocated to migrants by the authorities who facilitate returns do not provide sustainable income solutions in corrupt and economically unstable states. Furthermore, the widespread stigmatisation of returnees by their families and communities for failing long-term commitments pushes the former to re-migrate or change the place of residence within the borders of their states (Alpes, 2017; Eborka & Oyefara, 2016; Schuster & Majidi, 2015).

One of the best known ways to support rejected asylum-seekers or irregular migrants in return decisions is through the IOM-run Assisted Voluntary Return and Reintegration Program (hereafter, AVRR), of which VHR are a part. The scheme has been active since 1979 and the beneficiaries include rejected asylum-seekers, minors, stranded migrants and victims of trafficking. According to the IOM (2017), AVRR programmes are often the only solutions to the immediate plight of migrants. Criticising organised returns, Webber (2011: 104) has written that it is hardly possible to call them voluntary if the basic rights, support and work opportunities for migrants are otherwise denied. The situation of distress and acute danger can problematise volition even further. One of the responses of the IOM to the situation in the Libyan detention centres was to propose the return to migrants as an alternative to the inhumane and degrading conditions in the camps. This led to the new type of AVRR emerging at the end of 2017 (Alpes, 2020). The humanitarian aspect of the return operations transformed the narrative around clandestine migration. Irregular migrants in transit, who appeared to be at the centre of the restrictive EU migration policy, are now treated as victims whose well-being is under threat (Andersson, 2014: 68).

If returns are organised, the cooperation between ‘sending’ and ‘receiving’ states should be established and functioning. The Nigerian government has appeared to be one of the most approachable among West African states in organising the returns of its nationals. In addition, the Nigerian authorities were aware of the double EU agenda concerning returns. In 2017, President Buhari stated: ‘For people to cross the Sahara Desert and Mediterranean through shanty boats… we will try and keep them at home’ (BBC, 2017). Yet, what complicates such cooperation is the organisation of return from the territory of the third or transit state. To legitimise such arrangements outside territorial borders, the situation should be declared as ‘emergent’. Walters (2008) called similar strategies in terms of interventions ‘anti-policies’ or policies that are designed to combat negative occurrences such as terrorism, racism or trafficking in human beings. The fight against ‘dangerous’ and ‘problematic’ situations could ensue from the direct humanitarian actions on the spot as could the series of preventive activities. More precisely, to avoid victimisation in transit, migrants would be discouraged from embarking on the journey. Unsurprisingly, returnees who benefited from reintegration assistance became a valuable asset in testifying against taking the life-threatening Mediterranean route. The names of awareness-raising projects often speak for themselves; for example, the project ‘Migrants as messengers’^2^ which is currently running in Benin City. The ultimate goal of the programme is to spread the information about risks of illegal border crossings. Experimenting with different formats, such as theatre performances, radio programmes and outreach visits to schools and universities, returnees ‘testify’ to their experiences and discourage potential travellers from embarking on the dangerous journey to the EU. Thus, anti-policies ‘ultimately seek to discipline transnational movement by developing policies which aim to steer and organize the behaviour of individuals towards particular outcomes or decisions’ (Hastie, 2013: 126).

Reflecting on the absurdity of the attempts to tackle irregular migration, Andersson (2014: 40) has emphasised the entrepreneurial aspect of border management and called it an ‘illegal industry’. According to him, academics also take their share in the production of this industry. Among researchers-ethnographers, there is a tendency to ‘chase the crisis’ and, in so doing, although having good intentions, they might unwillingly reproduce victimising narratives and contribute to the further politicisation of returns (Cabot, 2019). Most of the academic research papers on facilitated returns discuss the lawfulness of such practices, the vulnerabilities of migrants or the challenges they faced upon arrival (see Alpes, 2020; Schuster & Majidi, 2015). Such contributions, although of tremendous value, are lacking the emphasis on returnees’ agency, which is an ultimate focus of this study.

## Formation and Performance of the Returnee Identity

There are different perspectives on how people label each other and self-identify (Jenkins, 1996, 2000). Sometimes, external categorisation serves as a basis for internal self-identification and vice versa. For example, Wood (1985) has described how certain political agendas create conditions for a new label to emerge. He has argued that ‘labelling is a way of referring to the process by which policy agendas are established and more particularly the way in which people, conceived as objects of policy are defined in convenient images’ (1985: 347).

Although the label is not always mentioned in the official documents, it could also emerge in the process of policy implementation. There is no precise definition of the ‘returnee’ category in EU migration legislation; nonetheless, ‘returnees’ are very often associated with the AVRR or VHR programmes and rarely with people who return through deportation. This is not surprising, since AVRR and VHR returns have a very clear ‘humanitarian’ element, giving the term ‘returnee’ a softer connotation compared to ‘deportee’ or ‘victim of trafficking in human beings’.

Assigned labels do not always correspond with how people would refer to themselves. For the identity to become ‘internalised’, there should be interaction between the labelling authorities and the individuals who are being labelled (Jenkins, 2000). Through interaction, labelled groups can change rules and transform the original definition, producing what Jenkins calls ‘mutual shifts in recognition’ (2000: 21). For instance, the category ‘returnee’, which was initially based on AVRRs and VHRs, could be modified and expanded to include people who are deported or who return by themselves and otherwise would not have benefited from the institutionalised assistance (Zetter, 1991, 2007).

The process of a label’s evolution and expansion correlates with the ‘policy translation’ phenomenon studied by development anthropologists who have looked at how aid policies were ‘translated’ and implemented by beneficiary states on a daily basis (Mosse, 2004; Mosse & Lewis, 2006). In many cases, such translations did not correspond with the initial agendas of the donors; instead, they gave an opportunity for beneficiaries to exercise agency and served as ‘an alternative way to succeed’ (Rodriguez, 2019: 746). As Olivier de Sardan (2005) has argued, Western (EU, US) projects are profitable for local communities; thus, such profitability makes certain categories especially desired by local governmental and non-governmental sectors (Harrell-Bond, 1986).

To illustrate the process of identity formation in the everyday reality of returnees and local institutions, I use the metaphor of a play, which will assist in constructing a holistic understanding of how EU policies to counter irregular migration serve as a basis for the emergence of the new ‘performed returnee identity’. Goffman (1956) wrote that individuals act or perform to make a certain impression or gain a specific response. Any theatrical performance requires a scene-setting and every scene, in turn, has its front and back stages. Both stages emerge in the result of the policies’ translation. Although repetitive, this performance can be transformed and censored through interaction with the audience (Butler, 1988, 1993). The front stage, according to Goffman (1956: 13), ‘functions in a general and fixed fashion to define the situation for those who observe the performance’, whereas the back stage can be discreet and less performative. In many ways, individuals will aim to control the impressions and filter the acts on the front stage; however, on the back stage, they reveal contradictions and discontinuities outside the script. The experience of acting on the back and front stages transforms returnees and contributes to the internalisation of their identity.

The aim of this article is not to explore the victimisation of returnees; yet, I do acknowledge that the ‘play’ as described above is partly based on the ‘victimisation’ or ‘humanitarian’ narrative. In no way do such occurrences undermine migrants’ horrific experiences in the detention centres; instead, they aim to illustrate how the emphasis on certain victimising aspects could become an opportunity to establish oneself. For example, Dunn (2008: 1601) has argued that ‘having attached the meaning “victims” to themselves, they now work to convince others to share this understanding and, with the help of others, they become eligible for sympathy, assistance, and other available resources’. Analysing contemporary asylum procedures, Fassin (2013) has also observed that ‘gender torment and sexual harassment receive favourable attention, arouse sympathy, raise little questioning, and, ultimately, often benefit from a positive assessment’ (2013: 49). Consequently, asylum-seekers or, in our case, returnees, could adjust their biographies according to our Western expectations. Reflecting on the origins of African subjectivity, Achille Mbembe (2008) has shown that, since the West is defining the subject, the idea behind African subjectivity automatically loses its meaning (Mbembe, 2008; Sithole, 2014).

Nonetheless, the available ‘sympathy, assistance and other resources’ made it beneficial for returnees to identify as such. In his famous book, *Stigma: Notes on the management of spoiled identity*, Goffman (1963) describes how individuals develop agency and become active strategists in the fight against discrimination. Individuals ‘steer or muddle their ways through difficult scenarios, turning “bad” into “less bad” circumstances’ (Long, 2001: 14). Research on facilitated returns confirms that returnees often experience stigma upon their return and try to find ways to reconstruct their ‘spoiled’ identity in order to regain appreciation and respect (Alpes, 2017; Schuster & Majidi, 2015). For example, taking inspiration from the performativity theories of Goffman (1956), Butler (1993) and others, Häkli and his colleagues (2017) wrote that ‘becoming a refugee’ constitutes ‘employment of their mundane political agencies, both challenging and reproducing the complex socio-political and socio-material relations that constitute the refugee regime’ (2017: 190).

Nevertheless, not all those who returned chose to interact with the authorities and participate in the performance. The reasons for not reaching out for help or assistance could vary from trauma, a lack of information or a mistrust of service-providers or returnee-led organisation leaders (Alpes, 2020). Therefore, the politicisation of return gave grounds for the returnee label to develop and acquire new political meanings but did not make all those who had returned, ‘returnees’.

## Methodology

This paper is based on ethnographic fieldwork in Benin City, Nigeria, between November 2019 and May 2020. The choice of the site was determined by the migration history to the EU of Benin inhabitants through the infamous Central Mediterranean Route (Malakooti, 2016). Since the ‘live’ data collection was terminated due to COVID-19 restrictions, the only plausible option to continue the research was to maintain daily digital contact with 10 of my respondents. The interaction not only included phone calls but also the daily submission of audio and video diaries (Ahlin & Li, 2019). In this way, traditional ethnography was replaced by digital and mobile forms — less tangible but no less valuable (De Bruijn et al., 2009; Sheller & Urry, 2016).

My pre-pandemic ethnographic encounters included participant observation in returnees’ homes, in shops and in government institutions, as well as through participation in awareness-raising events against irregular migration. The interviews with respondents had a somewhat unstructured character (Schapendonk, 2011). More precisely, respondents shared their stories in whichever way they felt comfortable with; often, the story-telling process was stretched over months, revealing new and important details along the way. Additionally, several semi-structured interviews with NGO and government representatives (7 interviews in total) were conducted in combination with ethnographic observations.

Since there is no institutional agreement on who is a returnee, those from Libya became the first group with whom I closely interacted. Gradually, my returnee circle expanded and included people who were deported from African states, had returned from the EU (the majority) and returned by themselves from Libya (over land).

Overall, I made contact with 35 returnees during my stay in Benin City but closely interacted with 10 returnees (five male and five female) in the following 8 months.

## Being Labelled or Self-Identifying as a Returnee in Benin City

My ethnographic journey started from enquiring about who the different local institutions labelled as returnees in order to understand how the ‘translation’ of return policies facilitated the establishment of the new category.

From the beginning, the returnee category was closely associated with the launch of VHRs from Libya. One of the Edo State Task Force Against Human Trafficking (hereafter, the Task Force) workers said: ‘I think we started calling them returnees after 2017’. Unsurprisingly, the institution itself was established in August 2017. However, many organisations had been assisting EU states in the organisation of AVRR that were targeting both ‘victims’ and ‘illegal’ migrants for decades. In 2017, all relevant NGOs and government leaders of Benin City were gathered in a general assembly to discuss the ‘returnee’ issue. The agenda of the meeting was to brainstorm about how to cooperate in providing returnees with reintegration services. ‘Actually, nothing really changed, just before we focused on victims of trafficking and now we propose the same services to returnees’ — the NGO worker who was present at the assembly affirmed.

The reasoning behind the VHR closely resembles that of anti-trafficking policies. Both are actively utilised in the extraterritorial migration management of the EU (Hastie, 2013). The anti-trafficking policies in Benin City consist of information campaigns and reintegration assistance for victims who returned from Europe through AVRR. Under cover of preventing the possible exploitation of young people, the aim of the policies was to discipline and control their migration aspirations (Hastie, 2013). While discussing the emergence of the returnee category with service-providers, I enquired whether there is any difference between a ‘victim of trafficking’ and a ‘returnee’. One of the government employees posited that victims of trafficking differ from returnees in terms of assistance, meaning the ‘need of more psychological care’. The rescue of migrants in Libya implied a clear victimising element; therefore, the response of local service-providers was the same as in the case of trafficking in human beings. Gradually, the return narrative replaced or interchanged with the long-established history of human trafficking in Benin City (Carling, 2005). Due to the overwhelming number of requests to the IOM, post-return projects were also carried out through subcontracting. Given the interest of NGOs in the implementation of return projects, returnees from Libya became what Harrell-Bond (1986) called the ‘desired’ category, which facilitated the labelling process. For example, one of the returnees told the employee of the Task Force that ‘We are returnees; therefore, all humanitarian help should go to us’. I have observed that the ways in which returnees addressed government employees were quite horizontal, informal and somewhat demanding. It made it clear that returnees were fully aware of how important their presence is in the implementation and allocation of humanitarian funds.

During my stay in Benin City, I came across five people who returned from Libya in 2017 by themselves. They took the route through the Sahara Desert, escaping violence and instability. A VHR returnee, Sunday, who introduced me to the group, gave them the idea to request reintegration assistance from the IOM. However, once in the IOM office, the group was told that the IOM did not have ‘empowering projects’ for people ‘like them’ (who returned by themselves). ‘Empowered’ in the development language of Benin City implied financial remuneration or tangible goods.

After their unsuccessful visit to the IOM, these migrants tried their luck with the Task Force. Compared to the IOM, the Task Force employees had a broader understanding of what can be included in the returnee category: they assisted deported people and victims of trafficking returned from Asian and African states, though the main and the most ‘valuable’ group of returnees were those from Libya. Once in the office, one of the employees asked the informal leader of the group: ‘Are you really a returnee?’. ‘Yes’, was the answer. ‘When did you return?’, the Task Force employee continued. ‘We returned in 2017’. The employee paused for a moment and then replied: ‘But you do not look like returnees’. When questioned why this was the case, a simple answer was given: ‘They know how to work and we do not help those who returned in 2017; it is too long ago’.

Meanwhile, other employees assisted Cristina, who was returned by the Federal Government of Nigeria and the IOM in 2017 but had never picked up her designated financial support ‘on arrival’ nor completed the reintegration training. She was pregnant and had many health issues. There was no doubt that Cristina was a returnee; therefore, she was given assistance. In the process of the external returnee categorisation, certain unwritten rules came into existence, making not all returnees ‘ideal’ or worthy of support (see Christie’s, 1986 ‘ideal victim theory’).

Although institutions did not acknowledge some migrants as returnees, the latter were still self-identifying as such, like the above-mentioned group. Interestingly, they only began to do so after encountering returnees whose homecoming was facilitated by the IOM, media professionals and myself. As was discussed earlier, the formation of the returnee identity always consists in ‘self-making’ and ‘being made’ (Jenkins, 2000). In addition, a person’s identity should be validated by others to acquire a certain value in society (Loseke, 1997). When the VHR returnee (Sunday) referred the mentioned group to me, his understanding of the returnee label was relatively broad. He first mentioned people returned by the IOM, then deportees and then people who had returned from Libya by themselves but, in the end, he said: ‘I have no idea who a returnee actually is anymore; you are the researcher, find out and help me’. Nevertheless, by including the group of self-organised ‘returnees’ in the returnee category, he made the first step in validating their identity. In this way, the meaning of the label expanded despite the lack of institutional support for this group of self-returned people. Moreover, it can be argued that I myself played a role in this category expansion through my involvement and questions on this matter, which further contributed to the external validation of this more-expansive notion of returnee-ness.

Besides media and Western researchers, local academics also shape the formation of the label. Upon my arrival in Nigeria, I was automatically classified as a returnee researcher by University of Benin employees and was proposed skilled assistance.^3^ Avoiding the path of ‘academic fixers’ or, in other words, intermediate agents who ‘secure’ and subsequently ‘control’ access to hard-to-reach populations, I decided to use the services of the assistants only once, mainly to understand who they would classify as returnees. As a result, I was taken to the home of three returnees from Libya. Two returned in 2018 through the IOM and the third returned by himself at the beginning of 2011 after the war in Libya had sparked. This last returnee received no formal assistance and was not on the Task Force’s database; nevertheless, his first words after he introduced himself were: ‘I am a returnee’.

Another way to validate one’s identity is through joining grassroots associations. Although such movements are often associated with autonomy and independence, very often, the authorities facilitate their establishment (Choudry & Kapoor, 2013). For example, the Task Force ‘empowered’ the formation of the ‘Go-getters’ movement, the idea behind which was to fight the stigma associated with return. According to one of the Task Force employees, we should look at returnees as dreamers, not losers.

One of the semi-formal activities which the go-getters perform is traffic control. More precisely, they assist the police in regulating traffic and receive a small stipend. Often, their services are used at official events and celebrations. When go-getters control the traffic, they wear bright vests produced by one of the returnees who successfully opened a tailoring workshop. During the COVID-19 lockdown, go-getters acquired the possibility to impose fines on those who were not wearing masks or who exceeded the maximum number of people allowed in the car, which gave them more power and status in the city. The rules for joining the group are quite loose and, according to the president, read as follows:

When you travel to any country through illegal migration and you were caught by the security agency of that country and you were not accepted by the government over there and you were deported back home – automatically this person becomes a returnee. We have all kinds of returnees from Libya, South Africa, Burkina Faso.

Thus, according to this grassroots-movement leader, the element of ‘force’ through deportation or expulsion and the consequent victimisation represent the key elements that allow a person to be labelled a returnee. By taking such a broad view on returns, the leader made it possible for more people to receive access to the returnee label than formal institutions and NGOs, thereby contributing to a broadening of the category’s definition.

Hence, self-identifying as a returnee and being a member of the group makes the label legitimate outside policy documents. As discussed earlier, both returnees and service-providers become ‘eligible for sympathy, assistance and other available resources’ (Dunn, 2008: 1606). Since returnees are indeed portrayed from the victimisation angle, their supporters intentionally or unintentionally create their own sets of rules and propositions about who to consider a victim — or, in our case, a returnee (Best, 1997).

## Performing the Returnee to Fight Stigma

The stigma associated with homecoming is well documented and known (e.g., Alpes, 2017; Schuster & Majidi, 2015). There are different strategies that returnees can employ to fight it, one of the most common of which is to re-migrate or hide the unsuccessful attempt from the family and community by changing the initial place of residence (Alpes, 2017; Schuster & Majidi, 2015). In the case of facilitated returns, returnees should publically acknowledge and embrace their new status, which logically makes it impossible to cover up their return.

A shining example is the story of Andrew. Upon his return in 2017, he came across a well-known Nigerian documentary filmmaker and asked to be accepted by the Benin Film Academy free of charge. The filmmaker was positive about the request and, shortly after, Andrew joined the course.

A: I also did a photography course and I did not pay – I told them I am a returnee.

Me: How does it feel to be a returnee?

A: Actually, people do not like returnees; they are treating them badly.

Me: But, as you told me last time, Nigerian people let you follow the course in cinematography because you are a returnee?

The reply followed a short moment of silence.

A: Actually, true. It is because I am a returnee that they allowed me to follow these courses for free; if I was not a returnee, they would not allow me to do it. I think I am actually privileged to be a returnee. I do volunteer work for the Edo State Task Force against Trafficking. For example, I got to know that there were food packages for returnees (...) If I were not a returnee, I would probably not achieve what I have achieved up to now. I actually benefited a lot from being a returnee. These benefits gave me hope. To be honest, I would not be able to study cinematography without being a returnee.

Andrew had also emphasised that he is ‘proud to be a returnee’ and that his mother is fully aware of his journey.

One of the most important elements in using a returnee identity to gain access to benefits is the emphasis on victimisation, which supposedly provokes compassion rather than judgement. During the COVID-19 lockdown in April 2020, Andrew started his own returnee association and actively collaborated with the IOM and the UNHCR. He is a good example of how people ‘steer or muddle their way through difficult scenarios, turning “bad” into “less bad” circumstances’ (Long, 2001: 14). Moreover, as noted by Taylor (2001), individuals use positive conceptions of stigma to achieve certain goals. Andrew made active use of his victimised narrative and gained recognition. He did it when applying to film and photography schools, when answering the formal questions of Western journalists and when he joined the IOM’s ‘theatre plays’ aimed at the discouragement of migration. However, the recognition and respect of his co-nationals did not influence his plans to re-migrate. Thus, Andrew was not only using his identity to fight stigma but was participating in the return migration play. Notably, returnees did not refer to themselves as such in many of their daily routines, which points to the performative nature of the returnee identity. ‘I think they are returnees with us — researchers, journalists, but not among themselves’, observed one Western journalist who was doing her assignment in Benin City.

Although all the returnees I encountered complained about the horrors of the journey towards Libya, not all reported ‘bad conditions’ on arrival. Both Andrew and Desmond (active members and leaders of returnee grassroots organisations) mentioned that they had no ‘big’ problems in Libya prior to the interception and detention that resulted from failed crossing attempts. ‘You have to be lazy not to have money in Libya; there is money in Libya’, Andrew told me. Yet, when we started discussing his professional returnee activities, he mentioned that Libya was ‘hell’ and he would do anything to stop people from experiencing what he had gone through. Desmond also told me that, although Libyans were very kind to him, he did not believe it was the case for everyone and felt ‘obliged’ to spread the word against irregular migration. He was also very content with his activities as secretary of the grassroots organisation that gave him the opportunity to work with Western partners and practise project-management skills.

If actors were not successful in performing the returnee identity, they were left with no choice but to search for other ways to succeed. Avoiding the judgement of his community in Benin City, Tim made the decision to stay in Lagos on his return from Libya. He had recently created a WhatsApp support group for returnees. During our call, Tim told me: ‘I know everything about the journey and can stop these people’ (from migrating). He asked me whether I was affiliated with the IOM, hoping to offer the organisation his services. It was a very difficult time for him; his business went down due to COVID-19 and awareness-raising activities seemed a possible way to ‘earn’ some respect and money. Meanwhile, people active in the same WhatsApp group informed me about their plans to undertake the journey to Libya again (including Tim). It was, eventually, either ‘to help the IOM to stop others’ or ‘to go to Libya again’.

I met Nella at one of the reintegration training courses for returned migrant women. She came back from Libya in 2017 with the assistance of her uncle. After a brief conversation, I asked whether we could meet again and she invited me to her house. Nella’s husband, a chief of the local community in the suburbs of the city, was fully aware of the unsuccessful journey of his wife; he was also informed about where Nella and I met. Welcoming a white person in the house for the first time made a big impression on the small community; therefore, the small living room was full of people. Being aware of my interest in return migration, Nella had also asked a local migration broker to join us. As a traveller and successful businessman, he was keen on sharing his experiences with his young neighbours and me. Despite the fact that his migration attempts had eventually failed, the community members treated him with great respect. At first, Nella avoided the conversation and was a bit hesitant about mentioning her return but, after a bit, she did share some details of her trip. Although she could not brag about the success of her ‘Libyan adventures’, the returnee label provided her with an opportunity to host a white foreigner. Thus, at that moment, Nella was not a failure — she was a big woman. This is a shining example of how communities which are normally more inclined to stigmatise returnees tend to embrace their new status after acknowledging some of its evident benefits — in this case, the privilege of hosting a white foreigner.

Furthermore, by following our research agendas, we (the researchers) created new acts in the script, subsequently contributing to returnees playing along with both pro- and counter-migration narratives. Consider the following conversation I had with Alex, the president of one of the returnees’ grassroots organisations.

A: They (international organisations, the IOM) are neutral, they do not understand the game and therefore can enter it any way they like. They apply for 2 million grants and we know that this is the money they demanded. So, the money is given to them by the EU. They get this money but the money they pass on to us is not enough to mobilise ourselves to do what we want with this money.

Me: But if you do not like the IOM, why do you volunteer for them?

A: Why do we not like them? We like them for their humanitarian services. We are not just condemning them because they are not good. We just need them to change. We want them to do the training on time. The conditions are too great for us. We need help.

To clarify, the IOM was, at the time, criticised by returnees and journalists for not providing the promised reintegration support in time. Additionally, the conditions for receiving such support (mainly in building up a small enterprise) were described as bureaucratic and difficult (see, for instance, Monello & Creta, 2020).

The prestige of being labelled as a returnee was linked to but not restricted by Western support. The leaders of the grassroots associations/movements expanded the number of followers and consequently became more visible on the political scene of Benin City. This made returnees deviate from following the initial EU-inspired script to fit the local politics of the city. For example, at the time of the Edo State elections, returnees were actively campaigning for the-then State governor, attending demonstrations and making official video campaigns. Additionally, returnees engaged in the social life of the city by organising talent shows (e.g. ‘Returnee has a talent’) and concerts and even directed TV programmes.^4^

## Hierarchies and the Unequal Distribution of Resources

Some returnees prospered through acting in the return-migration play, whereas others did not have the access nor the desire to participate in the various acts. For them, the returnee activists were further up the hierarchy: the latter were ‘ogas’,^5^ ‘big men’ and ‘sirs’. Power did not make returnee leaders less caring or supportive but such support was always linked to respect for and loyalty to their authority.

I witnessed the inner dynamics between returnee ‘activists’ and ‘other’ returnees quite often, but one situation best illustrates it. My Western friend and I, accompanied by Sunday and his friends, were having a drink in the park with Ben, the head of a returnee grassroots association. He arrived at the meeting point with his ‘press secretary’ and some of ‘his people’ who were roaming around the park. Sunday was the one introducing us to Ben because of his dream of establishing a grassroots organisation himself in the near future and was enthusiastic about meeting other returnees. Halfway through the conversation, Sunday asked Ben why the latter was working with the UNHCR since, according to Sunday, all international organisations were corrupt. The question made Ben very agitated.

B: Who said I am corrupt? You should not even be speaking, just sit quietly, as your friend. I am not corrupt, I do not get money from the UNHCR. I am a returnee! I have been to Libya!

S: I am also a returnee! I have also been to Libya and even got shot!

B: I am not going to talk to you. I came here to meet with (pointing to me and my friend) them, so you just have to stay silent. I do not even know you well; I met you once in the IOM office.

After this remark, Ben stood up and left the table. Sunday was confused; he said to us:

S: We are all returnees, why is he behaving like this? Why should I stay silent? I should not. Acting like a big man.

This case illustrates how the understanding of the returnee identity echoes the humanitarian narrative of victimisation discussed above. To be considered a ‘true’ returnee, one has to undergo the Libyan suffering. Yet, as part of a go-getter movement, one did not have to validate the suffering but, rather, be accepted by ‘returnee’ authorities. Leaders of grassroots movements were also respected by local government authorities (e.g. in the Task Force). While spending 2 days a week ‘working’ at the Nigerian Task Force against Trafficking of Human Beings, I had observed how closely the Task Force employees were interacting with returnees and how much say the returnee leaders had in, for instance, the distribution of humanitarian aid.

Reflecting on self-identification in the collective victimhood, Bar-Tal and his colleagues (2009) maintain that, apart from the evident traumatic experience, what emerges in the process of a victim’s identity formation is the need for empathy and recognition. Although the authors discussed collective victimhood in conflicts, the concept reflects the reality of returnees:

The sense of being a victim creates a sense of differentiation and superiority. It sharpens intergroup differences because, while it describes the opponent in delegitimizing terms and at the same time is responsible for the unjust and immoral acts, it presents its own society as a sole victim of the conflict (Bar-Tal et al., 2009: 243).

Simultaneously, the returnee identity became a source of capital and status accumulation which, in turn, impinged upon the establishment of hierarchies, as in the case of Ben and Sunday. Although the work of grassroots movements is essential and successful in fighting stigma, access to the returnee ‘status’ is not equally distributed.

## Conclusion

In this article, I have looked at how Voluntary Humanitarian Returns serve as a basis for the formation of the ‘returnee identity’. Identity formation is a complex and dynamic process. Thus, the profitability of being labelled as returnees and the lack of institutional definitions led to the expansion of the returnee category, which now includes that those who returned by themselves from Libya were deported from Europe or returned from other African states or Asia. In this evolution, one element remained stable — victimisation. One should either be deported, escape the violence or be returned by the authorities in order to be labelled or self-identify as a returnee. This victimising narrative was actively utilised in the interactions of returnees with different institutions, media and researchers. As suggested by Dunn (2008), ‘victims’ are convincing others about their victimisation so as to become eligible for assistance and sympathy. Consequently, it adds a performative element to the interactions between returnees, institutions and Westerners. Taking inspiration from Goffman’s metaphor of the play, we observe that, on the front stage, returnees are acting according to the script created by the restrictive EU migration management whereas, on the back stage, they use their identity to fight stigma and establish themselves in the city. The returnee identity, although initially a by-product of counter-migration initiatives, made some returnees become political actors who are setting their ‘rules’ and creating hierarchies. From just ‘returning migrants’, they became proud ‘returnees’. The narrative of suffering and compassion produced by Western authorities is the main source of their pride. However, the acting opportunity was not equally distributed among all returning migrants and, behind the scenes, returnee activists served as gatekeepers when it came to the possibilities that are brought along with the returnee label.

The return narrative is certainly being reinvented in Benin City which, on the one hand, assists in fighting the stigma associated with migration failure, giving migrants-deserved appreciation and a ‘voice’; however, on the other, the mechanisms leading to this appreciation contribute to what Andersson (2014) calls ‘absurd’. ‘Absurdity’, according to him, ‘covers a range of meanings, from the existential to the colloquial, but what will initially concern us here is the absurd in its guise of purposelessness pure and simple’ (2014: 349). Identity as such is not absurdist, especially considering its role in fighting stigma by giving the returnee identity a positive connotation; yet, the absurdity mainly concerns the play where this identity is performed. The EU migration-management policies, covered by ‘solely’ humanitarian purposes and adopted by local Nigerian actors, largely failed their initial purpose to reduce the numbers of people migrating to the EU and to address development issues. Instead, they resulted in a range of expensive performances. As EU-funded researchers, we definitely contribute to the existence of this play by framing our research around it. Perhaps, it is essential for future research projects to account for the performative element in the context of return and develop further understanding of what is happening behind the scenes.
